# 10-Bromo-*N*,*N*-di­phenyl­anthracen-9-amine

**DOI:** 10.1107/S2414314624002074

**Published:** 2024-03-12

**Authors:** K. Sureshkumar, Themmila Khamrang, Madhukar Hemamalini, Dhandayutham Saravanan, G. Jerald Maria Antony

**Affiliations:** a Department of Chemistry, National College, Thiruchirappalli, Tamil Nadu, India (Affiliated to Bharathidasan University, Tiruchirappalli); b Department of Chemistry, Dhanamanjuri University, Manipur 795 001, India; c Department of Chemistry, Mother Teresa Women’s University, Kodaikanal, Tamil Nadu, India; University of Aberdeen, United Kingdom

**Keywords:** crystal structure, hydrogen bonding

## Abstract

In the title compound, C_26_H_18_BrN, the dihedral angles between the anthracene ring system and the phenyl rings are 89.51 (14) and 74.03 (15)°. In the extended structure, a weak C—H⋯Br inter­action occurs, which generates [100] chains, but no significant π–π or C—H⋯π inter­actions are observed.

## Abstract 2

There are no significant π–π or C—H⋯π inter­actions in the extended structure of the title compound.

## Graphical abstract

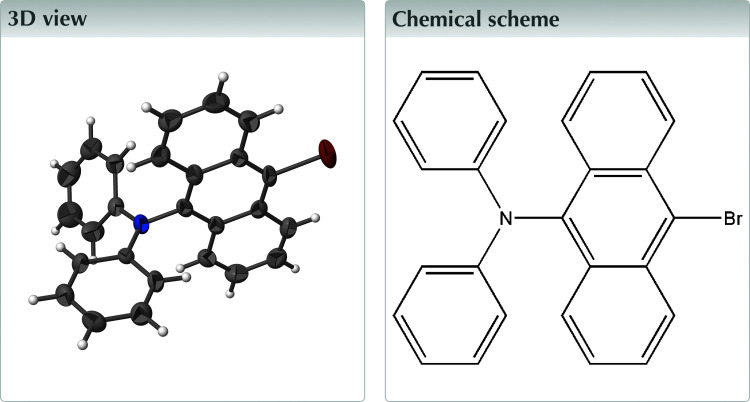

## Structure description

Palladium-catalysed cross-coupling reactions are an important method for the formation of various types of carbon–carbon and carbon–heteroatom bonds (Ruiz-Castilo & Buchwald, 2016 ▸). The anthracene nucleus is a key building block that has been extensively used in OLEDs and anion sensors, as well as electronic and optical materials (Dhangar *et al.*, 2017 ▸). As part of our work in this area, we now describe the structure of the title compound, C_26_H_18_BrN, (I).

**Figure 1:**
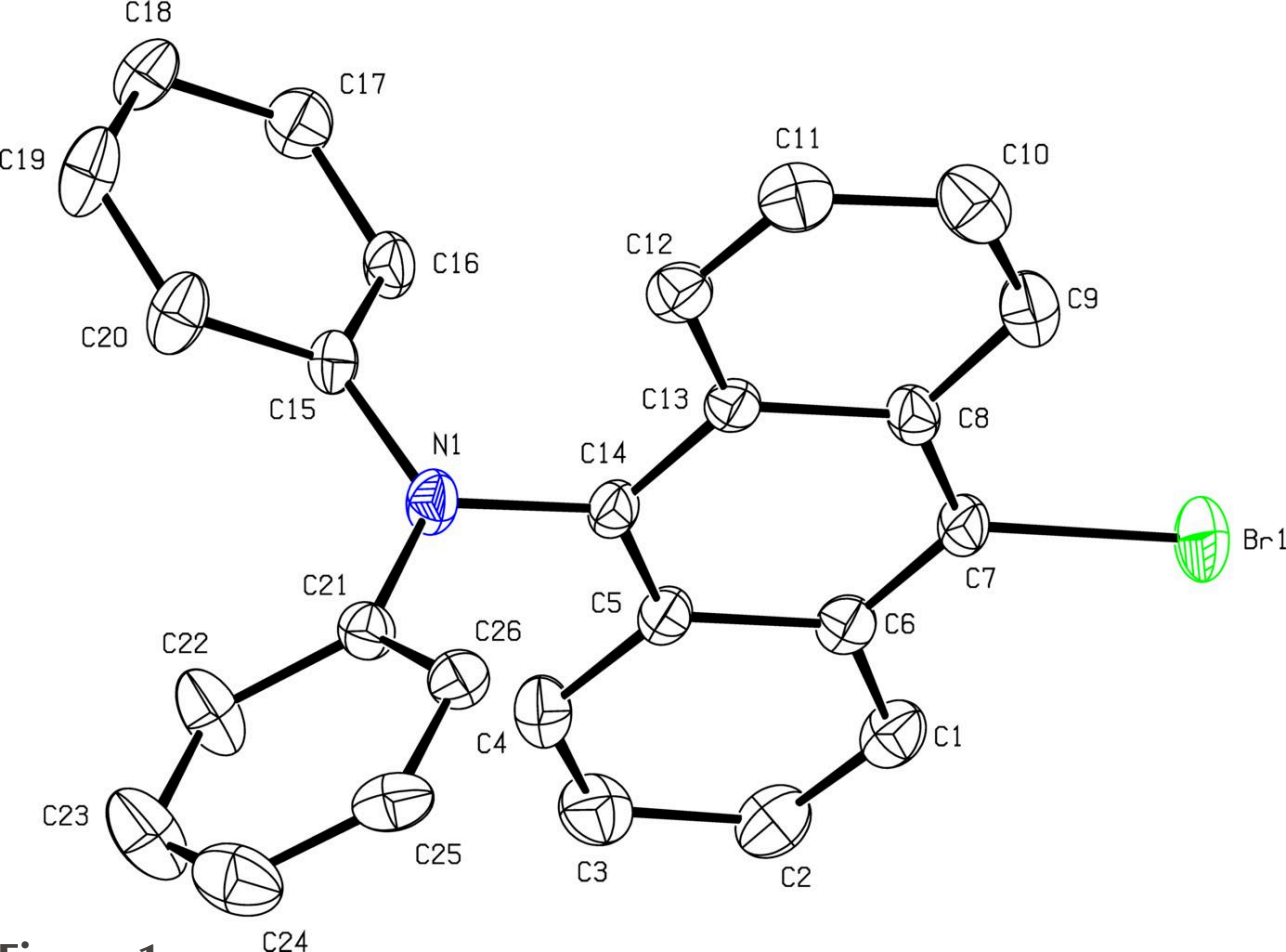
The asymmetric unit of (I). Displacement ellipsoids are drawn at the 50% probability level (H atoms are omitted for clarity).

**Figure 2:**
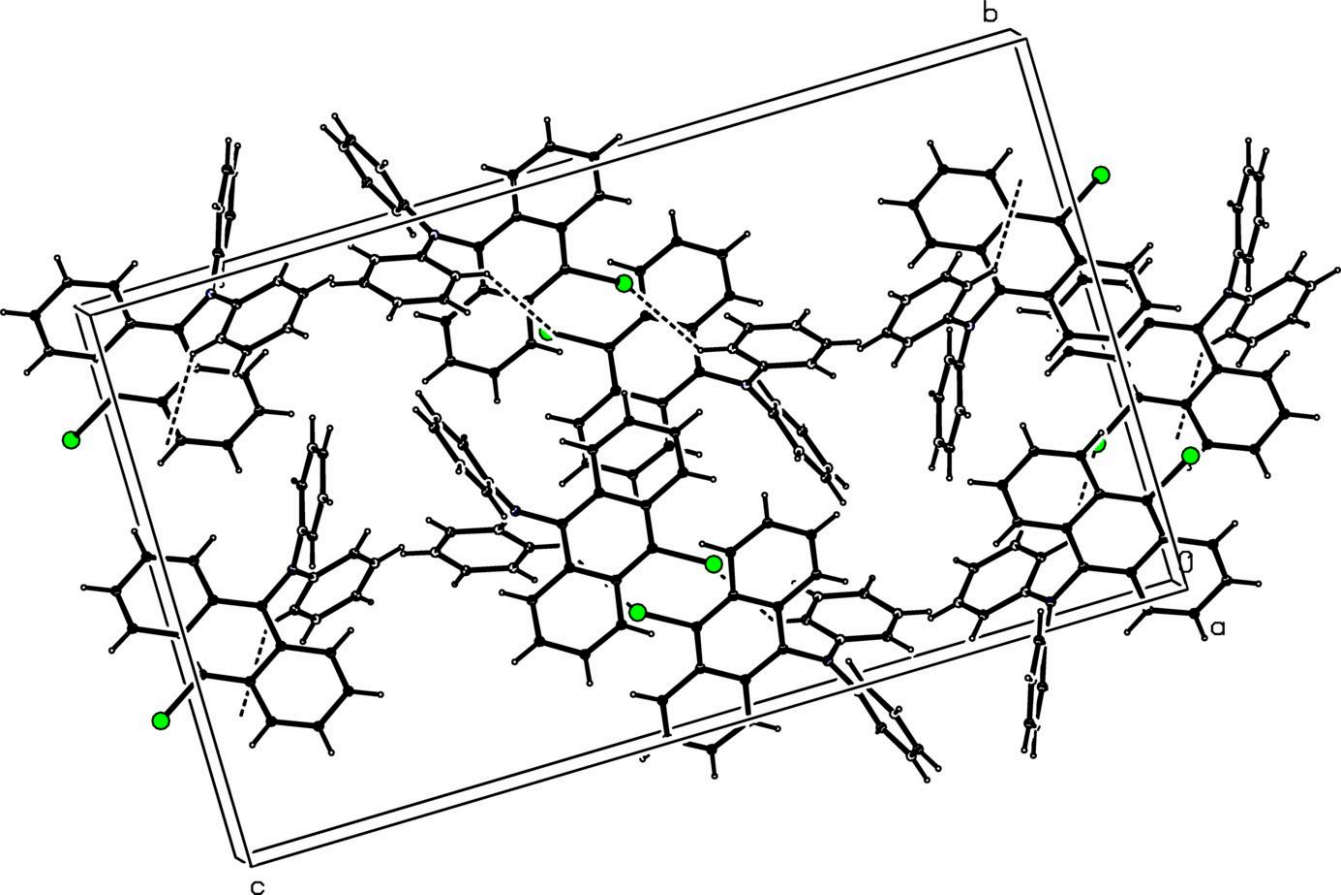
The crystal packing of the title compound.

The asymmetric unit of (I) is shown in Fig. 1 ▸: it crystallizes in space group *Pbca*. Compound (I) consists of a bromo-substituted anthracenyl moiety and two phenyl groups linked by the N atom. The compound is not planar as indicted by the dihedral angles between the anthracene ring system (C1–C14) and the phenyl rings (C15–C20 and C21–C26) of 89.51 (14) and 74.03 (15)°, respectively; the dihedral angle between the phenyl rings is 59.87 (19)°. The bond-angle sum at N1 is 360.0°. In the extended structure of (I) (Fig. 2 ▸), the only identified directional inter­action is a weak C16—H13⋯Br1 bond (Table 1 ▸), which generates [100] chains. No π–π or C—H⋯π inter­actions involving the aromatic rings occur.

A search of the Cambridge Structural Database (CSD; Version 5.41, update November 2019 (Groom *et al.*, 2016 ▸) for the 4-bromo­benzohydrazide fragment yielded many structures such as 10-bromo-2,7-di-*tert*-butyl-*N*,*N*-bis­(4-methyl­phen­yl) anthracen-9-amine (CSD refcode FEKTOG; Hoffend *et al.*, 2021 ▸), 10-bromo-*N*,*N*-bis­(4-methyl­phen­yl)anthracen-9-amine di­chloro­methane solvate (HOWJIO; Rajamalli *et al.*, 2015 ▸) and 9-(10′-bromo-9′-anthr­yl)carbazole (PEDSUM; Boyer *et al.*, 1993 ▸).

## Synthesis and crystallization

The title compound was synthesized as described previously (Justin Thomas *et al.*, 2005 ▸). Colourless blocks of (I) were recrystallized from the mixed solvents of di­chloro­methane and hexane.

## Refinement

Crystal data, data collection and structure refinement details are summarized in Table 2 ▸.

## Supplementary Material

Crystal structure: contains datablock(s) global, I. DOI: 10.1107/S2414314624002074/hb4462sup1.cif

Structure factors: contains datablock(s) I. DOI: 10.1107/S2414314624002074/hb4462Isup2.hkl

Supporting information file. DOI: 10.1107/S2414314624002074/hb4462Isup3.cml

CCDC reference: 2337417

Additional supporting information:  crystallographic information; 3D view; checkCIF report

## Figures and Tables

**Table 1:** Hydrogen-bond geometry (Å, °)

*D*—H⋯*A*	*D*—H	H⋯*A*	*D*⋯*A*	*D*—H⋯*A*
C16—H13⋯Br1^i^	0.93	2.92	3.621 (3)	133

Symmetry code: (i) 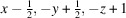 .

**Table 2:** Experimental details

Crystal data	
Chemical formula	C_26_H_18_BrN
*M*_r_	424.32
Crystal system, space group	Orthorhombic, *P* *b* *c* *a*
Temperature (K)	293
*a*, *b*, *c* (Å)	8.4890 (12), 16.400 (2), 27.936 (3)
*V* (Å^3^)	3889.3 (9)
*Z*	8
Radiation type	Mo *K*α
μ (mm^−1^)	2.13
Crystal size (mm)	0.37 × 0.32 × 0.29

Data collection	
Diffractometer	Agilent Xcalibur, Atlas, Gemini
Absorption correction	Multi-scan (*CrysAlis RED*; Agilent, 2012 ▸)
*T*_min_, *T*_max_	0.507, 0.578
No. of measured, independent and observed [*I* > 2σ(*I*)] reflections	9307, 3958, 2511
*R*_int_	0.044
(sin θ/λ)_max_ (Å^−1^)	0.625

Refinement	
*R*[*F* ^2^ > 2σ(*F* ^2^)], *wR*(*F* ^2^), *S*	0.053, 0.109, 1.08
No. of reflections	3958
No. of parameters	254
H-atom treatment	H-atom parameters constrained
Δρ_max_, Δρ_min_ (e Å^−3^)	0.32, −0.45

Computer programs: *CrysAlis PRO* (Agilent, 2012 ▸), *SHELXT2018/2* (Sheldrick, 2015*a* ▸), *SHELXL2018/3* (Sheldrick, 2015*b* ▸) and *PLATON* (Spek, 2020 ▸).

## full crystallographic data

### Crystal data

**Table:**

C_26_H_18_BrN	*D*_x_ = 1.449 Mg m^−^^3^
*M**_r_* = 424.32	Mo *K*α radiation, λ = 0.71073 Å
Orthorhombic, *P**b**c**a*	Cell parameters from 9307 reflections
*a* = 8.4890 (12) Å	θ = 3.5–26.4°
*b* = 16.400 (2) Å	µ = 2.13 mm^−^^1^
*c* = 27.936 (3) Å	*T* = 293 K
*V* = 3889.3 (9) Å^3^	Block, colourless
*Z* = 8	0.37 × 0.32 × 0.29 mm
*F*(000) = 1728	

### Data collection

**Table:**

Agilent Xcalibur, Atlas, Gemini diffractometer	2511 reflections with *I* > 2σ(*I*)
Radiation source: fine-focus sealed tube	*R*_int_ = 0.044
ω scans	θ_max_ = 26.4°, θ_min_ = 3.5°
Absorption correction: multi-scan (CrysAlis RED; Agilent, 2012)	*h* = −9→10
*T*_min_ = 0.507, *T*_max_ = 0.578	*k* = −20→20
9307 measured reflections	*l* = −34→33
3958 independent reflections	

### Refinement

**Table:**

Refinement on *F*^2^	Hydrogen site location: inferred from neighbouring sites
Least-squares matrix: full	H-atom parameters constrained
*R*[*F*^2^ > 2σ(*F*^2^)] = 0.053	*w* = 1/[σ^2^(*F*_o_^2^) + (0.0257*P*)^2^] where *P* = (*F*_o_^2^ + 2*F*_c_^2^)/3
*wR*(*F*^2^) = 0.109	(Δ/σ)_max_ = 0.001
*S* = 1.08	Δρ_max_ = 0.32 e Å^−^^3^
3958 reflections	Δρ_min_ = −0.45 e Å^−^^3^
254 parameters	Extinction correction: SHELXL-2018/3 (Sheldrick 2018), Fc^*^=kFc[1+0.001xFc^2^λ^3^/sin(2θ)]^-1/4^
0 restraints	Extinction coefficient: 0.0053 (3)
Primary atom site location: dual	

### Special details

**Table:**

Geometry. All esds (except the esd in the dihedral angle between two l.s. planes) are estimated using the full covariance matrix. The cell esds are taken into account individually in the estimation of esds in distances, angles and torsion angles; correlations between esds in cell parameters are only used when they are defined by crystal symmetry. An approximate (isotropic) treatment of cell esds is used for estimating esds involving l.s. planes.
Refinement. All the H atoms were positioned geometrically (C—H = 0.93 A°) and refined using a riding model with *U*_iso_(H) = 1.2 *U*_eq_(C).

### Fractional atomic coordinates and isotropic or equivalent isotropic displacement parameters (Å^2^)

**Table:**

	*x*	*y*	*z*	*U*_iso_*/*U*_eq_	
Br1	1.03399 (6)	0.27738 (3)	0.45892 (2)	0.0636 (2)	
N1	0.7773 (3)	0.45829 (17)	0.63518 (9)	0.0375 (7)	
C15	0.6336 (4)	0.43220 (19)	0.65522 (12)	0.0320 (8)	
C14	0.8370 (4)	0.4150 (2)	0.59378 (12)	0.0343 (8)	
C8	0.9751 (4)	0.2977 (2)	0.55873 (13)	0.0348 (9)	
C6	0.8719 (4)	0.4056 (2)	0.50658 (12)	0.0358 (8)	
C16	0.5297 (4)	0.3848 (2)	0.62905 (13)	0.0391 (9)	
H13	0.553092	0.372536	0.597332	0.047*	
C21	0.8715 (4)	0.5215 (2)	0.65491 (11)	0.0351 (8)	
C7	0.9532 (4)	0.3325 (2)	0.51364 (12)	0.0373 (9)	
C13	0.9143 (4)	0.3399 (2)	0.60012 (12)	0.0349 (8)	
C5	0.8115 (4)	0.4480 (2)	0.54798 (11)	0.0346 (8)	
C26	1.0326 (4)	0.5107 (2)	0.66114 (12)	0.0392 (9)	
H23	1.079724	0.462083	0.651752	0.047*	
C20	0.5938 (4)	0.4510 (3)	0.70287 (12)	0.0484 (10)	
H17	0.660305	0.483083	0.721415	0.058*	
C12	0.9361 (4)	0.3048 (2)	0.64666 (13)	0.0414 (9)	
H24	0.896293	0.331417	0.673439	0.050*	
C9	1.0548 (4)	0.2222 (2)	0.56713 (15)	0.0490 (10)	
H27	1.094870	0.193335	0.541178	0.059*	
C1	0.8415 (4)	0.4411 (2)	0.46022 (12)	0.0453 (10)	
H6	0.880669	0.415718	0.432949	0.054*	
C17	0.3931 (4)	0.3554 (2)	0.64880 (14)	0.0486 (10)	
H14	0.326265	0.322997	0.630532	0.058*	
C4	0.7233 (4)	0.5212 (2)	0.54110 (13)	0.0445 (9)	
H3	0.683742	0.549023	0.567501	0.053*	
C19	0.4559 (4)	0.4214 (3)	0.72170 (14)	0.0567 (12)	
H16	0.430422	0.434191	0.753200	0.068*	
C18	0.3540 (4)	0.3736 (2)	0.69575 (13)	0.0505 (10)	
H15	0.261406	0.353978	0.709322	0.061*	
C3	0.6966 (4)	0.5506 (2)	0.49646 (14)	0.0534 (10)	
H4	0.637559	0.597837	0.492550	0.064*	
C11	1.0136 (4)	0.2338 (2)	0.65207 (15)	0.0500 (10)	
H25	1.027900	0.212476	0.682598	0.060*	
C10	1.0736 (5)	0.1912 (3)	0.61196 (16)	0.0559 (11)	
H26	1.126037	0.142003	0.616256	0.067*	
C25	1.1231 (5)	0.5716 (2)	0.68114 (13)	0.0506 (10)	
H22	1.230573	0.563347	0.685389	0.061*	
C2	0.7577 (4)	0.5101 (3)	0.45570 (14)	0.0526 (11)	
H5	0.739522	0.531558	0.425392	0.063*	
C22	0.8063 (5)	0.5955 (2)	0.66815 (14)	0.0603 (12)	
H19	0.699330	0.604758	0.663454	0.072*	
C24	1.0571 (6)	0.6439 (3)	0.69480 (17)	0.0678 (14)	
H21	1.118793	0.684722	0.708352	0.081*	
C23	0.8986 (6)	0.6554 (3)	0.68822 (18)	0.0793 (15)	
H20	0.852705	0.704461	0.697454	0.095*	

### Atomic displacement parameters (Å^2^)

**Table:**

	*U*^11^	*U*^22^	*U*^33^	*U*^12^	*U*^13^	*U*^23^
Br1	0.0805 (4)	0.0629 (3)	0.0473 (3)	0.0032 (2)	0.0161 (2)	−0.0246 (3)
N1	0.0372 (16)	0.0429 (18)	0.0323 (15)	−0.0014 (15)	0.0035 (15)	−0.0111 (15)
C15	0.0292 (18)	0.0375 (19)	0.0293 (17)	0.0071 (16)	−0.0006 (17)	−0.0035 (17)
C14	0.0286 (18)	0.039 (2)	0.0358 (19)	−0.0064 (16)	0.0069 (17)	−0.0093 (17)
C8	0.0312 (19)	0.034 (2)	0.039 (2)	−0.0035 (15)	0.0003 (18)	−0.0106 (18)
C6	0.0305 (18)	0.042 (2)	0.0352 (18)	−0.0103 (17)	−0.0018 (17)	−0.0076 (18)
C16	0.044 (2)	0.040 (2)	0.0335 (19)	−0.0001 (18)	0.0032 (19)	−0.0130 (18)
C21	0.042 (2)	0.0332 (18)	0.0300 (18)	−0.0009 (17)	−0.0006 (18)	−0.0016 (16)
C7	0.040 (2)	0.040 (2)	0.0317 (19)	−0.0088 (17)	0.0050 (18)	−0.0150 (18)
C13	0.0354 (19)	0.035 (2)	0.0347 (18)	−0.0113 (16)	0.0047 (18)	−0.0073 (17)
C5	0.0319 (19)	0.0396 (19)	0.0323 (18)	−0.0043 (17)	0.0041 (17)	−0.0048 (18)
C26	0.044 (2)	0.038 (2)	0.035 (2)	0.0000 (18)	0.0015 (19)	0.0026 (18)
C20	0.037 (2)	0.076 (3)	0.0315 (18)	−0.001 (2)	−0.0016 (19)	−0.013 (2)
C12	0.042 (2)	0.044 (2)	0.037 (2)	−0.0102 (18)	−0.0008 (19)	−0.0031 (19)
C9	0.046 (2)	0.049 (2)	0.053 (2)	0.0047 (19)	0.000 (2)	−0.014 (2)
C1	0.044 (2)	0.058 (2)	0.0338 (19)	−0.010 (2)	0.001 (2)	−0.006 (2)
C17	0.046 (2)	0.053 (2)	0.047 (2)	−0.006 (2)	−0.001 (2)	−0.008 (2)
C4	0.039 (2)	0.048 (2)	0.047 (2)	0.0048 (19)	0.001 (2)	−0.010 (2)
C19	0.045 (2)	0.089 (3)	0.036 (2)	0.007 (2)	0.006 (2)	−0.012 (2)
C18	0.034 (2)	0.066 (3)	0.052 (2)	−0.002 (2)	0.013 (2)	0.001 (2)
C3	0.050 (2)	0.048 (2)	0.062 (3)	0.009 (2)	−0.004 (2)	0.008 (2)
C11	0.050 (2)	0.053 (3)	0.047 (2)	0.002 (2)	−0.006 (2)	0.008 (2)
C10	0.047 (2)	0.051 (3)	0.070 (3)	0.009 (2)	−0.012 (2)	−0.004 (2)
C25	0.049 (2)	0.055 (2)	0.048 (2)	−0.018 (2)	−0.004 (2)	0.009 (2)
C2	0.050 (2)	0.068 (3)	0.041 (2)	−0.002 (2)	−0.003 (2)	0.007 (2)
C22	0.058 (3)	0.045 (2)	0.078 (3)	0.011 (2)	−0.015 (2)	−0.023 (2)
C24	0.081 (4)	0.053 (3)	0.070 (3)	−0.019 (3)	−0.016 (3)	−0.005 (3)
C23	0.091 (4)	0.049 (3)	0.097 (4)	0.006 (3)	−0.014 (4)	−0.025 (3)

### Geometric parameters (Å, º)

**Table:**

Br1—C7	1.903 (3)	C12—H24	0.9300
N1—C15	1.409 (4)	C9—C10	1.360 (5)
N1—C21	1.420 (4)	C9—H27	0.9300
N1—C14	1.449 (4)	C1—C2	1.342 (5)
C15—C16	1.385 (4)	C1—H6	0.9300
C15—C20	1.408 (4)	C17—C18	1.386 (5)
C14—C5	1.406 (4)	C17—H14	0.9300
C14—C13	1.406 (5)	C4—C3	1.356 (5)
C8—C7	1.395 (5)	C4—H3	0.9300
C8—C9	1.431 (5)	C19—C18	1.374 (5)
C8—C13	1.443 (4)	C19—H16	0.9300
C6—C7	1.398 (5)	C18—H15	0.9300
C6—C1	1.443 (4)	C3—C2	1.416 (5)
C6—C5	1.444 (4)	C3—H4	0.9300
C16—C17	1.371 (5)	C11—C10	1.415 (5)
C16—H13	0.9300	C11—H25	0.9300
C21—C22	1.384 (4)	C10—H26	0.9300
C21—C26	1.390 (5)	C25—C24	1.366 (5)
C13—C12	1.434 (5)	C25—H22	0.9300
C5—C4	1.428 (5)	C2—H5	0.9300
C26—C25	1.378 (5)	C22—C23	1.376 (5)
C26—H23	0.9300	C22—H19	0.9300
C20—C19	1.372 (5)	C24—C23	1.372 (6)
C20—H17	0.9300	C24—H21	0.9300
C12—C11	1.345 (5)	C23—H20	0.9300
			
C15—N1—C21	123.7 (3)	C8—C9—H27	119.0
C15—N1—C14	118.1 (3)	C2—C1—C6	121.3 (4)
C21—N1—C14	118.1 (3)	C2—C1—H6	119.3
C16—C15—C20	118.0 (3)	C6—C1—H6	119.3
C16—C15—N1	120.8 (3)	C16—C17—C18	120.5 (4)
C20—C15—N1	121.2 (3)	C16—C17—H14	119.7
C5—C14—C13	121.6 (3)	C18—C17—H14	119.7
C5—C14—N1	118.9 (3)	C3—C4—C5	120.6 (3)
C13—C14—N1	119.5 (3)	C3—C4—H3	119.7
C7—C8—C9	124.4 (3)	C5—C4—H3	119.7
C7—C8—C13	118.7 (3)	C20—C19—C18	122.4 (3)
C9—C8—C13	116.9 (3)	C20—C19—H16	118.8
C7—C6—C1	124.2 (3)	C18—C19—H16	118.8
C7—C6—C5	118.4 (3)	C19—C18—C17	118.1 (4)
C1—C6—C5	117.4 (3)	C19—C18—H15	120.9
C17—C16—C15	121.6 (3)	C17—C18—H15	120.9
C17—C16—H13	119.2	C4—C3—C2	120.8 (4)
C15—C16—H13	119.2	C4—C3—H4	119.6
C22—C21—C26	118.1 (3)	C2—C3—H4	119.6
C22—C21—N1	121.2 (3)	C12—C11—C10	120.9 (4)
C26—C21—N1	120.7 (3)	C12—C11—H25	119.5
C8—C7—C6	122.9 (3)	C10—C11—H25	119.5
C8—C7—Br1	118.9 (3)	C9—C10—C11	120.2 (4)
C6—C7—Br1	118.1 (3)	C9—C10—H26	119.9
C14—C13—C12	121.7 (3)	C11—C10—H26	119.9
C14—C13—C8	119.0 (3)	C24—C25—C26	120.9 (4)
C12—C13—C8	119.2 (3)	C24—C25—H22	119.5
C14—C5—C4	121.8 (3)	C26—C25—H22	119.5
C14—C5—C6	119.3 (3)	C1—C2—C3	120.9 (4)
C4—C5—C6	118.9 (3)	C1—C2—H5	119.6
C25—C26—C21	120.5 (3)	C3—C2—H5	119.6
C25—C26—H23	119.8	C23—C22—C21	120.5 (4)
C21—C26—H23	119.8	C23—C22—H19	119.8
C19—C20—C15	119.4 (4)	C21—C22—H19	119.8
C19—C20—H17	120.3	C25—C24—C23	119.0 (4)
C15—C20—H17	120.3	C25—C24—H21	120.5
C11—C12—C13	120.8 (4)	C23—C24—H21	120.5
C11—C12—H24	119.6	C24—C23—C22	121.0 (4)
C13—C12—H24	119.6	C24—C23—H20	119.5
C10—C9—C8	121.9 (4)	C22—C23—H20	119.5
C10—C9—H27	119.0		
			
C21—N1—C15—C16	164.7 (3)	N1—C14—C5—C6	179.4 (3)
C14—N1—C15—C16	−19.2 (5)	C7—C6—C5—C14	0.4 (5)
C21—N1—C15—C20	−17.6 (5)	C1—C6—C5—C14	179.3 (3)
C14—N1—C15—C20	158.5 (3)	C7—C6—C5—C4	−177.7 (3)
C15—N1—C14—C5	98.7 (4)	C1—C6—C5—C4	1.1 (5)
C21—N1—C14—C5	−84.9 (4)	C22—C21—C26—C25	1.7 (5)
C15—N1—C14—C13	−79.2 (4)	N1—C21—C26—C25	−178.8 (3)
C21—N1—C14—C13	97.2 (4)	C16—C15—C20—C19	0.6 (5)
C20—C15—C16—C17	−1.1 (5)	N1—C15—C20—C19	−177.2 (3)
N1—C15—C16—C17	176.6 (3)	C14—C13—C12—C11	−178.4 (3)
C15—N1—C21—C22	−48.2 (5)	C8—C13—C12—C11	0.6 (5)
C14—N1—C21—C22	135.6 (4)	C7—C8—C9—C10	179.6 (4)
C15—N1—C21—C26	132.2 (3)	C13—C8—C9—C10	−0.3 (5)
C14—N1—C21—C26	−43.9 (4)	C7—C6—C1—C2	177.6 (3)
C9—C8—C7—C6	178.6 (3)	C5—C6—C1—C2	−1.2 (5)
C13—C8—C7—C6	−1.4 (5)	C15—C16—C17—C18	0.9 (6)
C9—C8—C7—Br1	−0.2 (5)	C14—C5—C4—C3	−178.2 (3)
C13—C8—C7—Br1	179.7 (2)	C6—C5—C4—C3	0.0 (5)
C1—C6—C7—C8	−177.1 (3)	C15—C20—C19—C18	0.1 (6)
C5—C6—C7—C8	1.6 (5)	C20—C19—C18—C17	−0.3 (6)
C1—C6—C7—Br1	1.7 (4)	C16—C17—C18—C19	−0.2 (6)
C5—C6—C7—Br1	−179.5 (2)	C5—C4—C3—C2	−1.0 (6)
C5—C14—C13—C12	−178.0 (3)	C13—C12—C11—C10	−0.9 (6)
N1—C14—C13—C12	−0.2 (5)	C8—C9—C10—C11	0.1 (6)
C5—C14—C13—C8	2.9 (5)	C12—C11—C10—C9	0.6 (6)
N1—C14—C13—C8	−179.2 (3)	C21—C26—C25—C24	−0.6 (6)
C7—C8—C13—C14	−0.8 (5)	C6—C1—C2—C3	0.2 (6)
C9—C8—C13—C14	179.1 (3)	C4—C3—C2—C1	0.9 (6)
C7—C8—C13—C12	−179.9 (3)	C26—C21—C22—C23	−1.9 (6)
C9—C8—C13—C12	0.0 (5)	N1—C21—C22—C23	178.5 (4)
C13—C14—C5—C4	175.4 (3)	C26—C25—C24—C23	−0.2 (7)
N1—C14—C5—C4	−2.5 (5)	C25—C24—C23—C22	−0.1 (7)
C13—C14—C5—C6	−2.7 (5)	C21—C22—C23—C24	1.1 (7)

### Hydrogen-bond geometry (Å, º)

**Table:**

*D*—H···*A*	*D*—H	H···*A*	*D*···*A*	*D*—H···*A*
C16—H13···Br1^i^	0.93	2.92	3.621 (3)	133

Symmetry code: (i) *x*−1/2, −*y*+1/2, −*z*+1.
